# The Call to Members of the College

**Published:** 1919-06-28

**Authors:** 


### THE CALL TO MEMBERS OF THE COLLEGE.

Sir Arthur Stanley's Manchester address marks an epoch in the history of nursing. It was a very remarkable record of achievement, set forth with perfect clearness, and with characteristic modesty. There was no beating of drums or blowing of trumpets, and I am not quite sure whether this is a matter for regret or congratulation. I cannot help wondering how many of his hearers realised the full force of the Chairman's facts and figures. Probably not a very large percentage. The majority of us are more or less dazed by the magnitude and the number of startling occurrences, and, consequently, miracles of achievement are apt to be regarded as commonplace. I speak feelingly, and as one of the dazed. The greater wonder is not the progress of the College, on which Sir Arthur Stanley dwelt, but rather the concept and creation of this great institution, of which he said nothing. Looking backward, it seems quixotic to have started such an enterprise in the throes of a great war, and at a time when most people held their breath with anxiety, when enemy hosts were advancing in millions to rend this Empire limb from limb. The founder of the College did not pause to call all this to memory, but quietly observed that "the College only came into being at the end of 1915," leaving the rest to his hearers' imagination and intelligence.

### The Feeling of Neglect.

Every nurse has for years suffered from a feeling of neglect, and in a sense she is right. And yet I have no doubt that a generation hence the British nurse will arrive at a different conclusion. She will feel convinced that, not after the war, but in the midst of the storm, her interests were being championed. She will learn that out of chaos, and in the teeth of fierce domestic opposition and faction fighting, a wondrous edifice was erected. Please note, in passing, the word "domestic." No opposition to the College has come from the public, but from a disgruntled and self-seeking clique. I entertain no doubt that the nurse of the next generation will regard the opposition now engaged in composing hymns of hate precisely as we look back to-day with pity and contempt on poor old Sarah Gamp. But it was ever thus. Even the Church thundered against the use of chloroform when it was first discovered, and no doubt Sarah had her influential supporters.

If it is not an impertinence, I venture to suggest that the College of Nursing should print and circulate in leaflet form Sir Arthur Stanley's Manchester address, and that every one of its fourteen thousand members should keep a copy in her pocket-book. In the absence of this, a leaf out of last week's Hospital (pp. 297 and 298) will be a good substitute. In making this suggestion I am not inspired by sentimental considerations. It is a matter of business. The address contains in tabloid an extraordinary collection of extraordinary facts. Out of the void, in extremely difficult and trying times, the College was created. So excellent was its design that within three years fourteen thousand trained nurses have joined as members. Over £40,000 stands to their credit, to be expended for their benefit. A College State Registration Bill is at present before Parliament. Nineteen local centres for the social and professional betterment of nurses have been established and are flourishing. An admirable system of scholarships for providing higher professional training for the rank and file has been founded. And last, but by no means least, an economic department has been set up for standardising and improving nurses' conditions of employment.

### A Well-Ordered Establishment.

It is my misfortune to be a critic. Thereby I make many enemies. Truth is not always a welcome visitor, and criticism consists in sifting the truth from the chaff. And, speaking critically, I regard this record as the story of a miracle. But it is not merely because of its being a fascinating story that I want the nurse to have it in her pocket-book. I have a distinctly utilitarian object in view. Sir Arthur Stanley made only one request at Manchester, and he made it in the same tone that he announced the achievements of the College. I do not know whether, like Lord Clive on a certain historic occasion, he stands amazed at his own modesty. There are those of us who do. Sir Arthur asks for very little, and his request is to the nurse, and in her interests. Let this not be forgotten or overlooked. It is all in her interests, every atom. In the Book of Lies the modern Sarah Gamp tells fantastic tales of "gold bugs," of "employers," of men and women of title seeking to put the independent nurse into chains and consigning her to slavery. The College has bitter enemies, and what the Chairman points out is this, that if certain detailed and undeniable achievements are on record, greater triumphs will be won if there is a membership of twenty thousand. If the members of the College are wise in their generation they will see that their Chairman's call is answered, immediately and without delay. If fourteen thousand have joined in blind faith and out of the chaos, forty thousand at least should join on the evidence of things seen and tangible. The fourteen thousand pinned their faith to a speculation. They were sports. New recruits are invited to enter a well-ordered establishment. In the early stages there was no house, but merely an architect's drawing. Now, the house is built, and there is money in the bank, even forty thousand pounds. What has the house of Bedlam to offer the nurse? I have tried to find out, and I have failed, but I may be ignorant, and if there is any information of which I am unaware I shall be glad to receive it.

### The College Member's Duty.

Meanwhile the plain duty of the College member is to go forth as a vigorous and enthusiastic recruiting officer. This duty she owes to her College and her chief, and it is to enable her to be effective that I suggest she should be provided with the necessary equipment. She will be asked, "What is your College? What has it done? What does it hope to accomplish?" The answers are wrapt up in Sir Arthur Stanley's Manchester address. The College carries in its origin and constitution the soul of Florence Nightingale, the most modest, the most persevering, the most enlightened, and, withal, the most determined pioneer of effectual and efficient nursing that England has ever produced. I have seen her on a cinema screen. I have read of her in many books but I am convinced that from the standpoint of the rank and file her life's work is by no means fully known or appreciated. The mantle of Florence Nightingale has fallen on Sir Arthur Stanley, and it is the bounden duty of every College member to see that his call for recruits is not regarded as a trivial consideration. Membership and articulation are one and the same thing, and therefore the most practical method for the nurse to express herself is to respond to the call.

IERNE.

